# Supplementary material for: Generation of interspecies limited chimeric nephrons using a conditional nephron progenitor cell replacement system
Source: Nat Commun. 2017 Nov 23;8:1719. doi: 10.1038/s41467-017-01922-5 (PMC5701015; doi:10.1038/s41467-017-01922-5)
Supplement: Supplementary file 2 — Description of Additional Supplementary Files [file 41467_2017_1922_MOESM2_ESM.pdf]

File Name: Supplementary Movie 1
Description: Movie showing confocal fluorescence micrographs presented in Figure 1-e. Red indicates Six2 (nephron progenitor marker). White indicates cytokeratin-8 (ureteric bud and collecting duct marker). Green indicates transplanted GFP-NPCs. The renal vesicle showed a mosaic structure in which the transplanted cells were green, and the host cells were not visible.

File Name: Supplementary Movie 2
Description: Observation of Six2-iDTR mouse embryos (E13.5) under a stereoscopic microscope. Nephron progenitor cells collected from CAG-DsRed mice were injected into the nephrogenic zone by mouth pipetting or using an injector device.

File Name: Supplementary Movie 3
Description: Movie showing confocal fluorescence micrographs presented in Figure 3-e-1. Red indicates WT1 (glomerular marker). Blue indicates cytokeratin-8 (ureteric bud and collecting duct marker). Green indicates transplanted GFP-NPCs.

File Name: Supplementary Movie 4
Description: Movie showing confocal fluorescence micrographs presented in Figure 3-e-2. Red indicates WT1 (glomerular marker). Blue indicates E-cadherin (distal tubule and collecting duct marker). Green indicates transplanted GFP-NPCs. White indicates DAPI.

File Name: Supplementary Movie 5
Description: Movie showing confocal fluorescence micrographs presented in Figure 3-e-3. Red indicates WT1 (glomerular marker). White indicates cytokeratin-8 (ureteric bud and collecting duct marker). Green indicates transplanted GFP-NPCs. Transplanted NPCs attached to the host cap mesenchyme and differentiated nephrons. The movie shows the differentiation of nephrons connected to the host ureteric bud.

File Name: Supplementary Movie 6
Description: Movie showing confocal fluorescence micrographs presented in Figure 3-e-4. White indicates cytokeratin-8 (ureteric bud and collecting duct marker). Green indicates transplanted GFP-NPCs. Transplanted NPCs attached to the host cap mesenchyme and differentiated nephrons. The movie shows the differentiation of nephrons connected to host ureteric bud.

File Name: Supplementary Movie 7
Description: Movie showing confocal fluorescence micrographs presented in Figure 4-d-2. Red indicates CD31 (endothelial marker). Blue indicates podocine (podocyte marker). Green indicates transplanted GFP-NPCs.

File Name: Supplementary Movie 8

Description: Movie showing confocal fluorescence micrographs presented in Figure 5-b. Red indicates Six2 (nephron progenitor marker). White indicates cytokeratin-8 (ureteric bud and collecting duct marker). Green indicates transplanted rat GFP-NPCs. Blue indicates DAPI.

File Name: Supplementary Movie 9

Description: Movie showing the drug-induced cell elimination system.
